# *Plagiorchis* sp. in small mammals of Senegal and the potential emergence of a zoonotic trematodiasis

**DOI:** 10.1016/j.ijppaw.2019.02.003

**Published:** 2019-02-14

**Authors:** Stefano Catalano, Steven A. Nadler, Cheikh B. Fall, Kirsty J. Marsh, Elsa Léger, Mariama Sène, Simon L. Priestnall, Chelsea L. Wood, Nicolas D. Diouf, Khalilou Bâ, Joanne P. Webster

**Affiliations:** a Centre for Emerging, Endemic and Exotic Diseases, Department of Pathobiology and Population Sciences, The Royal Veterinary College, University of London, Hatfield, AL97TA, UK; b Department of Entomology and Nematology, University of California, Davis, CA, 95616, USA; c Faculté de Médecine, de Pharmacie et d’Odonto-Stomatologie, Université Cheikh Anta Diop, Dakar, BP 5005, Senegal; d Unité de Formation et de Recherche des Sciences Agronomiques, d’Aquaculture et de Technologies Alimentaires, Université Gaston Berger, Saint-Louis, BP 234, Senegal; e School of Aquatic and Fishery Sciences, University of Washington, Seattle, WA, 98195, USA; f Centre de Biologie et de Gestion des Populations, Institut de Recherche pour le Développement, Dakar, BP 1386, Senegal; g London Centre for Neglected Tropical Disease Research, School of Public Health, Faculty of Medicine, Imperial College London, London, W21PG, UK

**Keywords:** *Plagiorchis*, Parasite, Trematode, West Africa, Wildlife, Zoonoses

## Abstract

Trematodes of the genus *Plagiorchis* have a wide geographical distribution and can exploit a variety of hosts. The occurrence and zoonotic potential of *Plagiorchis* spp. have been characterised across several countries in Asia; in contrast, information on *Plagiorchis* parasites in Africa remains anecdotal. We isolated a previously undescribed *Plagiorchis* species from the biliary tract and small intestine of 201 out of 427 small mammals collected in the region of Lake Guiers, Senegal, with local prevalence ranging from 38.6% to 77.0%. Conversely, *Plagiorchis* isolates were not observed in the 244 small mammals sampled in and around the town of Richard Toll, Senegal. Molecular phylogenetics of the internal transcribed spacer region, nuclear ribosomal DNA, and of the cytochrome *c* oxidase subunit 1 gene, mitochondrial DNA, supported the monophyly and multi-host spectrum of this newly discovered West African *Plagiorchis* species. Sequencing of individual cercariae shed by *Radix natalensis* (Gastropoda: Lymnaeidae) suggested that these freshwater snails may act as suitable first intermediate hosts. Phylogenetic analysis yielded a highly resolved topology indicating two different clades, one composed by *Plagiorchis* spp. infecting rodents, insectivores, and birds, while the other included parasites of bats. Our findings showed the low host specificity and high prevalence of the isolated *Plagiorchis* sp. in the Lake Guiers region, with Hubert's multimammate mice (*Mastomys huberti*) appearing to play a primary role in the epidemiology of this parasite. The results raise concern about the zoonotic potential of *Plagiorchis* sp. in local communities of the Lake Guiers region, and highlight food-borne trematodiases and their link to land-use change as a neglected public health issue in regions of West Africa.

## Highlights

• Monophyletic, previously undescribed *Plagiorchis* lineage in African small mammals.
• High local prevalence ranging from 38.6% to 77.0% in the examined hosts.
• Identification of the gastropod *Radix natalensis* as first intermediate host.
• Potential role of *Plagiorchis* sp. as emerging zoonotic pathogen in northern Senegal.

## Graphical abstract

**Figure:**
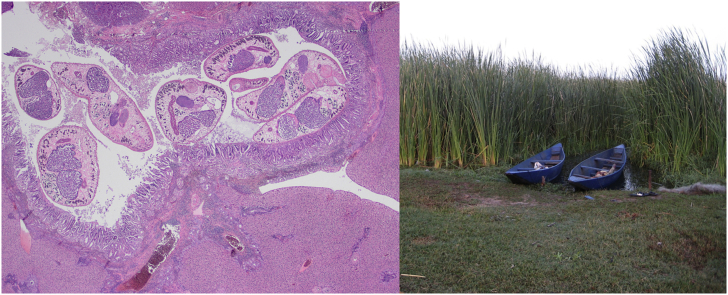

## 1 Introduction

Trematodes of the genus *Plagiorchis* have a heteroxenous life cycle involving freshwater pulmonate gastropods of the family Lymnaeidae as first intermediate hosts and mosquito and chironomid larvae, insect naiads, freshwater fish, and crustaceans as second intermediate hosts (Guk et al., 2007; Boyce et al., 2014; Soldánová et al., 2017). *Plagiorchis* spp. are cosmopolitan, their vast distribution spanning from boreal to tropical zones around the globe. Their wide geographic range may be a consequence of their relatively low host specificity, with records of adult parasites of the genus *Plagiorchis* from the small intestine of a variety of definitive hosts, including reptiles, birds, and mammals (Guk et al., 2007; Boyce et al., 2014).

*Plagiorchis* parasites also have documented zoonotic potential: *Plagiorchis muris* has been diagnosed to cause intestinal infections in human patients in Japan and the Republic of Korea (Asada et al., 1962; Hong et al., 1996). Intestinal infections of humans have also been observed for *Plagiorchis vespertilionis*, usually considered a bat parasite, in the Republic of Korea (Guk et al., 2007), and for *Plagiorchis harinasutai*, *Plagiorchis philippinensis*, and *Plagiorchis javensis* in Southeast Asia (Sandground, 1940; Radomyos et al., 1989, 1998). Further findings of *P. harinasutai*, *P. philippinensis*, and *P. javensis* seem sporadic and localised in the same countries where these *Plagiorchis* spp. were first described, with few human cases and one report on *P. harinasutai* infecting rodents in the Republic of the Philippines (Radomyos et al., 1998; Eduardo and Lee, 2006). However, the taxonomic validity of these records may be questionable since the identification of *Plagiorchis* isolates was based solely on morphological traits, which lack diagnostic accuracy due to their high intra-specific variability (e.g., Brendow, 1970; Tkach et al., 2000; Boyce et al., 2014; Zikmundová et al., 2014).

To our knowledge, *P. muris* is the only species isolated in mammalian hosts across the African continent after studies on helminths of black rats (*Rattus rattus*) in Nigeria (Udonsi, 1989; Ivoke, 2009). We focused on a region of West Africa where trematodiases have become of great public health concern as a consequence of anthropogenic land-use change. Epidemiological and molecular data on *Plagiorchis* parasites occurring in Hubert's multimammate mice (*Mastomys huberti*), Nile grass rats (*Arvicanthis niloticus*), and shrews (*Crocidura* sp.) of the Senegal River Basin were analysed, and a phylogenetic framework was developed to explore lineage diversity, host spectrum, and ultimately zoonotic potential of *Plagiorchis* isolates.

## 2 Materials and methods

We conducted our study in and around the town of Richard Toll and on the shores of Lake Guiers, Senegal. This region has experienced major infrastructure development (i.e., the completion of the Diama Dam in 1986) and land-use change that have generated a rapid agro-industrial growth while dramatically altering the ecology of the territory (Uhlir, 2003). These anthropogenic changes have been associated with outbreaks of schistosomiasis disease (a neglected tropical disease caused by *Schistosoma* trematodes), which is currently endemic in the Senegal River Basin and for which wild rodents may act as local reservoirs (Duplantier and Sène, 2000; Catalano et al., 2018). Sampling sites were geolocated, categorised based on habitat type (Supplementary Table S1), and grouped into four geographic areas: RT_1 (including six sites) in and around Richard Toll; and LG_1 (including seven sites), LG_2 (including seven sites), and LG_3 (including eight sites) on the shores of Lake Guiers (Supplementary Fig. S1). The shortest distance between two sites within the same area was approximately 500 m, whereas distances between two sites of different areas were >4 km.

Between May 2016 and December 2017, we trapped, humanely euthanized, and necropsied small mammals in compliance with ethical guidelines and methodologies previously described (Catalano et al., 2018). For each individual, we recorded species, anatomical measurements, gender, and sexual development. Rodents were classified as juveniles or adults based on the combination of body weight and reproductive status: *A. niloticus* and *M. huberti* with weight ≥ 70 g and ≥ 33 g, respectively, and developed sexual traits were considered adults (Granjon and Duplantier, 2009; Herbreteau et al., 2011). At post-mortem examination, thoracic and abdominal organs were visually inspected for helminths using tap water and a glass tray against a black background. Due to time constraints, the gastrointestinal tract was analysed for a randomly-selected subset of the hosts. Isolated trematodes were characterised to the genus level based on their morphology using an Olympus CX41 microscope (following Boyce et al., 2014), counted to determine infection intensity (counts stopped at 61 individuals due to time constraints, therefore higher intensities are referred to as > 61 and the value 62 was used in statistical analyses), and preserved in 95% ethanol at −20 °C. Samples of liver from infected and uninfected *M. huberti* were preserved in 10% neutral-buffered formalin followed by routine processing for histopathological examination. As part of a wide research programme conducted in the Senegal River Basin (http://www.theupstreamalliance.org), trematode cercarial stages shed by freshwater snails *Radix natalensis* (Gastropoda: Lymnaeidae) from our sampling areas were collected onto Whatman FTA^®^ indicator cards (GE Healthcare, Little Chalfont, UK).

DNA from adult *Plagiorchis* specimens was extracted using the QIAGEN DNeasy^®^ Blood & Tissue Kit (QIAGEN, Hilden, Germany) following the manufacturer's instructions. DNA from individual cercariae stored on indicator cards was extracted as previously described (Webster et al., 2015). DNA extracts were amplified for the complete internal transcribed spacer (ITS) region of the nuclear ribosomal DNA (rDNA), and for a segment of the cytochrome *c* oxidase subunit 1 (*cox1*) gene of the mitochondrial DNA (mtDNA), using the primers ETTS1 and ETTS2 (Kane and Rollinson, 1994), and 2575 and 3021 (Bowles et al., 1992), respectively. Enzymatic amplification for polymerase chain reaction (PCR) was performed in 25 μL reaction mixtures including PuReTaq™ Ready-To-Go™ PCR Beads (GE Healthcare, Little Chalfont, UK), 0.5 μM of each primer, and 2 μL of DNA template. Cycling parameters for the ITS region consisted of an initial nucleic acid denaturation at 95 °C for 5 min, followed by 35 cycles of 95 °C for 30 s, 56 °C for 1 min, and 72 °C for 1 min, with a final 7 min extension at 72 °C. Cycling parameters for the *cox1* gene consisted of an initial nucleic acid denaturation at 94 °C for 5 min, followed by 35 cycles of 94 °C for 30 s, 52 °C for 1 min, and 72 °C for 1 min, with a final 7 min extension at 72 °C. PCR products were sequenced using the original PCR primers in a 3730xl DNA Analyzer system by GATC Biotech (Konstanz, Germany). Assembly and editing of contigs were performed with CodonCode Aligner v8.0.1 (CodonCode Corporation, Centerville, MA, USA). These data, together with previously published ITS and *cox1* sequences available in the GenBank™ database (Table 1), were aligned using ProAlign v0.5 (Löytynoja and Milinkovitch, 2003). For the ITS alignment, the minimum posterior probability of sites was used as the criterion for detecting and removing unreliably aligned characters; the filter threshold was set to 60% minimum posterior probability (i.e., an intermediate value between the threshold of posterior probabilities for correctly versus incorrectly aligned sites) in order to reduce the likelihood of excluding correctly aligned sites (Löytynoja and Milinkovitch, 2003). The ITS and *cox1* alignments were analysed separately to infer phylogenetic relationships, executing Maximum Likelihood (ML) and Bayesian Inference (BI) on the Cyberinfrastructure for Phylogenetic Research (CIPRES) web portal (http://www.phylo.org) using RAxML v8.2 (Stamatakis, 2014) and MrBayes v3.2.6 (Ronquist et al., 2012), respectively. For ML analyses, best-fit evolutionary models (GTR model with invariable sites (+I) and rate heterogeneity (+G) for the *cox1* dataset, and GTR + G model for the ITS dataset) were selected using PartitionFinder v2.1.1 (Lanfear et al., 2016), with automatic arrest of bootstrap resampling to assess nodal support. For BI analyses, we invoked best-fit evolutionary models and partitioning schemes (i.e., GTR + G for ITS, GTR + I + G for *cox1* position one, F81 + I for *cox1* position two, GTR + I + G for *cox1* position three) using PartitionFinder v2.1.1 (Lanfear et al., 2016). Each BI analysis was performed using two independent runs with four Markov Chain Monte Carlo (MCMC) chains and four million generations. MCMC chains were sampled every 4000 generations and the first 25% of the trees was discarded as burn-in. The trees remaining after burn-in were used to create a 50% majority-rule consensus tree with posterior probabilities indicating nodal support. For both ML and BI, the resulting tree topologies were visualized using FigTree v1.4.3 (http://tree.bio.ed.ac.uk/software/figtree/).

**Table 1:** List of taxa used in our study, including their life cycle stage (A for adult, C for cercaria, and MC for metacercaria), sampling locality, host species, and GenBank™ accession numbers for the internal transcribed spacer (ITS), nuclear ribosomal DNA, and the cytochrome *c* oxidase subunit 1 (*cox1*), mitochondrial DNA.

*Plagiorchis* species	Stage	Location	Host	Accession no.	Sequence (base pairs)	Reference
*Plagiorchis* sp.	C	Norway	*Radix balthica*	KY513237-52	*cox1* (423)	Soldánová et al. (2017)
*Plagiorchis* sp.	C	Norway	*Gammarus lacustris*	KY513253-4	*cox1* (423)	Soldánová et al. (2017)
*Plagiorchis* sp.	C	Norway	*Radix balthica*	KY513255-7	*cox1* (423)	Soldánová et al. (2017)
*Plagiorchis* sp.	MC	Norway	*Tipula salicetorum*	KY513258	*cox1* (423)	Soldánová et al. (2017)
*Plagiorchis* sp.	C	Norway	*Radix balthica*	KY513259-60	*cox1* (423)	Soldánová et al. (2017)
*Plagiorchis* sp.	C	Norway	*Radix balthica*	KY513261-2	*cox1* (423)	Soldánová et al. (2017)
*Plagiorchis* sp.	C	Norway	*Radix balthica*	KY513263	*cox1* (423)	Soldánová et al. (2017)
*Plagiorchis* sp.	MC	Germany	*Lepidostoma* sp.	KX160474	ITS (1506)	Grabner (2017)
*Plagiorchis* sp.	MC	Germany	*Hydropsyche* sp.	KX160477	ITS (1476)	Grabner (2017)
*Plagiorchis* sp.	MC	Germany	*Paraleptophlebia* sp.	KX160478	ITS (1418)	Grabner (2017)
*P. maculosus*	C	Czech Republic	*Lymnaea stagnalis*	KJ533390-1	ITS (1072–1092)	Zikmundová et al. (2014)
*P. elegans*	C	Slovakia	*Lymnaea stagnalis*	KJ533399-404	*cox1* (423)	Zikmundová et al. (2014)
*P. elegans*	C	Czech Republic	*Lymnaea stagnalis*	KJ533405-16	*cox1* (423)	Zikmundová et al. (2014)
*P. koreanus*	C	Czech Republic	*Radix auricularia*	KJ533417-18	*cox1* (423)	Zikmundová et al. (2014)
*P. maculosus*	C	Czech Republic	*Lymnaea stagnalis*	KJ533419-28	*cox1* (423)	Zikmundová et al. (2014)
*P. neomidis*	C	Slovakia	*Lymnaea stagnalis*	KJ533429-35	*cox1* (423)	Zikmundová et al. (2014)
*Plagiorchis* sp.	C	Czech Republic	*Lymnaea stagnalis*	KJ533436	*cox1* (423)	Zikmundová et al. (2014)
*P. elegans*	A	England	*Apodemus sylvaticus*	JX522536	ITS (1213)	Boyce et al. (2014)
*P. maculosus*	A	Ukraine	*Fringilla coelebs*	AF316152	ITS (1100)	Snyder and Tkach (2001)
*P. koreanus*	A	Ukraine	*Pipistrellus kuhli*	AF151944	ITS (1193)	Tkach et al. (2000)
*P. koreanus*	A	Ukraine	*Nyctalus noctula*	AF151945	ITS (1193)	Tkach et al. (2000)
*P. koreanus*	A	Ukraine	*Myotis daubentoni*	AF151946	ITS (1193)	Tkach et al. (2000)
*P. muelleri*	A	Ukraine	*Eptesicus serotinus*	AF151947-8	ITS (1263)	Tkach et al. (2000)
*P. vespertilionis*	A	Ukraine	*Myotis daubentoni*	AF151949-51	ITS (1266)	Tkach et al. (2000)
*P. elegans*	A	Ukraine	*Lanius collurio*	AF151952	ITS (1232)	Tkach et al. (2000)
*P. muris*	MC	Republic of Korea	*Sympetrum* sp.	AF096236	*cox1* (443)	Lee et al. (2004)
*Plagiorchis* sp.	C	Senegal	*Radix natalensis*	MH633862	ITS (1159)	Our study
*Plagiorchis* sp.	C	Senegal	*Radix natalensis*	MH673682	*cox1* (369)	Our study
*Plagiorchis* sp.	A	Senegal	*Arvicanthis niloticus*	MH633857	ITS (1159)	Our study
*Plagiorchis* sp.	A	Senegal	*Arvicanthis niloticus*	MH673677	*cox1* (363)	Our study
*Plagiorchis* sp.	A	Senegal	*Crocidura* sp.	MH633858-9	ITS (1159)	Our study
*Plagiorchis* sp.	A	Senegal	*Crocidura* sp.	MH673678-9	*cox1* (396)	Our study
*Plagiorchis* sp.	A	Senegal	*Mastomys huberti*	MH633855-6	ITS (1159)	Our study
*Plagiorchis* sp.	A	Senegal	*Mastomys huberti*	MH633860-1	ITS (1159)	Our study
*Plagiorchis* sp.	A	Senegal	*Mastomys huberti*	MH673675-6	*cox1* (396)	Our study
*Plagiorchis* sp.	A	Senegal	*Mastomys huberti*	MH673680-1	*cox1* (396)	Our study

We applied a binomial generalized linear model (GLM) to explore the potential association between occurrence of *Plagiorchis* sp. (included as the dichotomous response variable), and the categorical explanatory variables of host species, gender, age class (excluding *Crocidura* sp.), and sampling area. We selected the cauchit link function as it improved model fit relative to other link functions (Koenker and Yoon, 2009); starting with the full model, we used the Akaike information criterion for model selection and step-wise deletion of non-significant terms. Variations in *Plagiorchis* sp. occurrence between biliary tract and intestine as the main infection site were explored using Pearson's chi-squared (χ^2^) test. Data on the intensity of infections showed a non-normal distribution; therefore, we explored differences in *Plagiorchis* sp. intensity between biliary tract and intestine using the non-parametric Wilcoxon signed-rank test for paired data. Statistical tests, significant when *P* ≤ 0.05, were implemented in R v3.1.2 (https://www.r-project.org).

## 3 Results

**Fig. 1:**
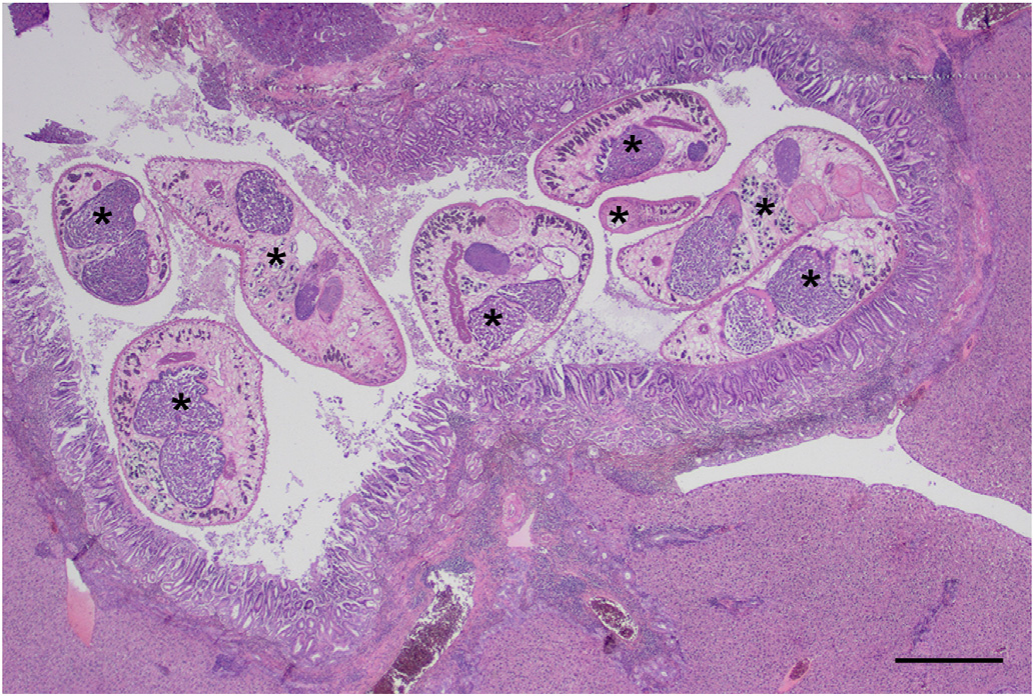
Histological section of liver from a Hubert's multimammate mouse (*Mastomys huberti*). A large central bile duct is markedly dilated by the presence of *Plagiorchis* trematodes (indicated by an asterisk). Marked hyperplasia of the lining biliary epithelium is shown, associated with moderate to marked lymphoplasmacytic cholangitis and mild to moderate lymphoplasmacytic hepatitis of the surrounding portal areas. Scale bar = 500 μm.

We set 4149 traps with effective capture rates of 15.4% in RT_1, 21.2% in LG_1, 13.6% in LG_2, and 15.7% in LG_3. Overall, we captured 671 small mammals, isolating *Plagiorchis* trematodes in 189 out of 324 *M. huberti* (58.3%), 7 out of 22 *Crocidura* sp. (31.8%), and 5 out of 81 *A. niloticus* (6.2%) from sites surrounding Lake Guiers. Among the surveyed areas, we observed a local prevalence of 38.6% in LG_1, 40.4% in LG_2, and 77.0% in LG_3 when considering the host community as a whole. In contrast, *Plagiorchis* sp. was not observed in the 244 small mammals captured in RT_1 (Table 2; for capture rates and parasitological analyses at each sampling site see Supplementary Table S2). Histopathological examination revealed trematode parasites morphologically consistent with *Plagiorchis* sp. within the bile ducts and, less frequently, portal veins. The infections were associated with marked hyperplasia of the biliary epithelium, moderate to marked lymphoplasmacytic cholangitis, and mild to moderate portal/periportal hepatitis (Fig. 1). The degree of parasite burden, as recorded histologically, generally corresponded to the severity of inflammation and biliary hyperplasia.

**Table 2:** Prevalence, intensity median and range of *Plagiorchis* sp. in Nile grass rats (*Arvicanthis niloticus*), Hubert's multimammate mice (*Mastomys huberti*), shrews (genus *Crocidura*), and gerbils (genus *Taterillus*) trapped in four areas around the town of Richard Toll (RT_1) and on the shores of Lake Guiers (LG_1, LG_2, and LG_3), Senegal.

Area	Organ	*A. niloticus*		*M. huberti*		*Crocidura*	*Taterillus*
		Juveniles	Adults	Juveniles	Adults		
RT_1	Liver	0/62	0/114	0/19	0/24	0/19	0/6
	Intestine	0/14	0/57	0/8	0/18	–	–
LG_1	Liver	0/7	0/39	4/19 (21.1%) 14, 3-26	61/102 (59.8%) 13, 1->61	7/22 (31.8%) >61, 5->61	–
	Intestine	0/1	0/22	1/13 (7.7%) 19	15/76 (19.7%) 5, 1-21	0/14	–
LG_2	Liver	0/1	4/27 (14.8%) 1.5, 1-37	14/36 (38.9%) 22, 3->61	39/87 (44.8%) 16, 1->61	–	–
	Intestine	0/1	0/27	0/21	10/72 (13.9%) 3.5, 1-26	–	–
LG_3	Liver	–	1/7 (14.3%) >61	14/25 (56.0%) 42, 7->61	49/55 (89.1%) >61, 1->61	–	–
	Intestine	–	0/7	6/25 (24.0%) 3.5, 1-8	38/55 (69.1%) 9, 1->61	–	–

**Fig. 2:**
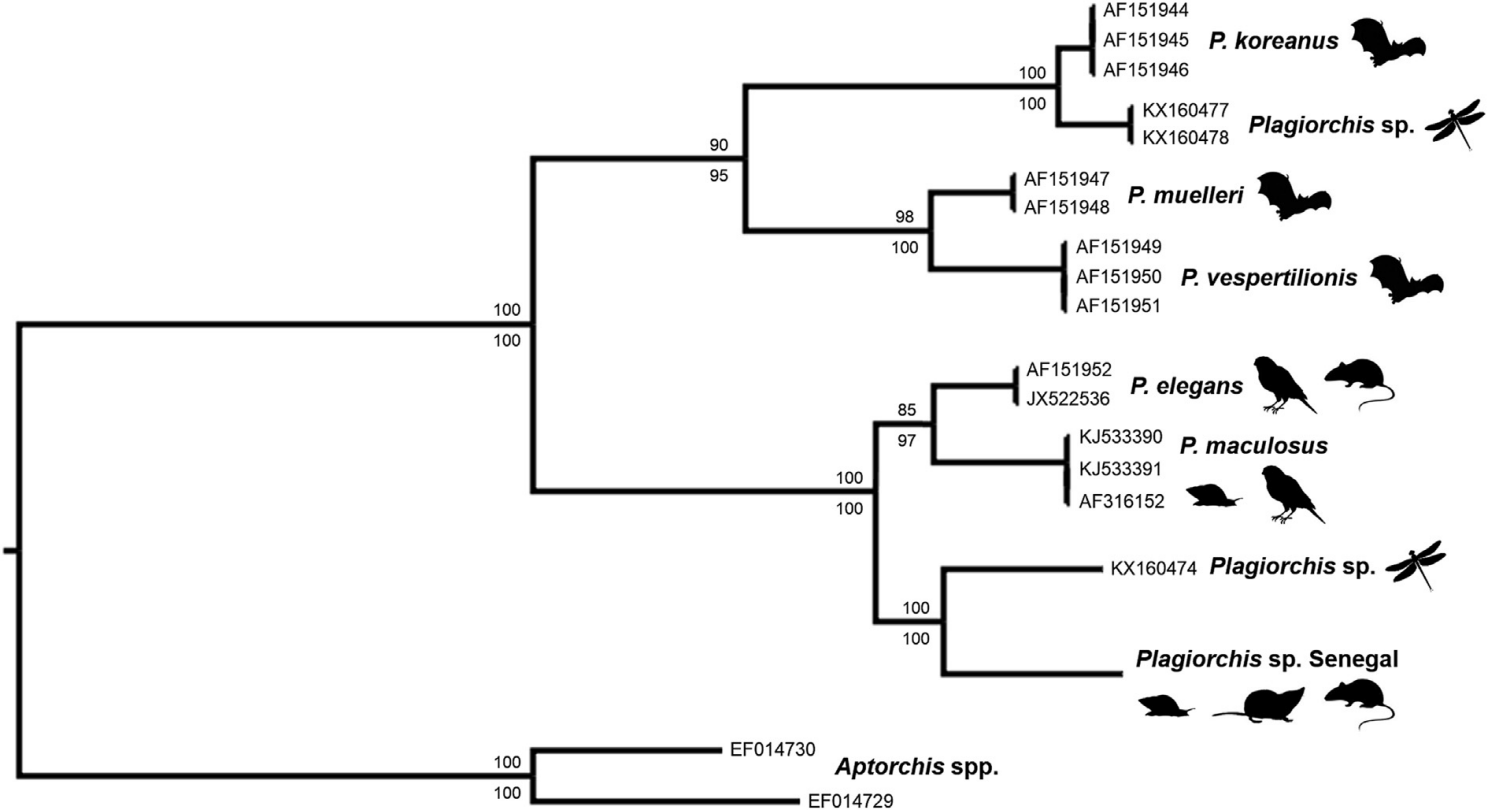
Phylogenetic relationships among *Plagiorchis* spp. inferred by Maximum Likelihood (ML) and Bayesian Inference (BI) analyses of the internal transcribed spacer sequence data. The black silhouettes represent the hosts from which the molecular data of *Plagiorchis* spp. were obtained. The taxa *Aptorchis aequalis* and *Aptorchis megacetabulus* (GenBank™ EF014729 and EF014730, respectively) were used as outgroups. Nodal support is indicated as ML percentage above and BI posterior probability below each branch.

**Fig. 3:**
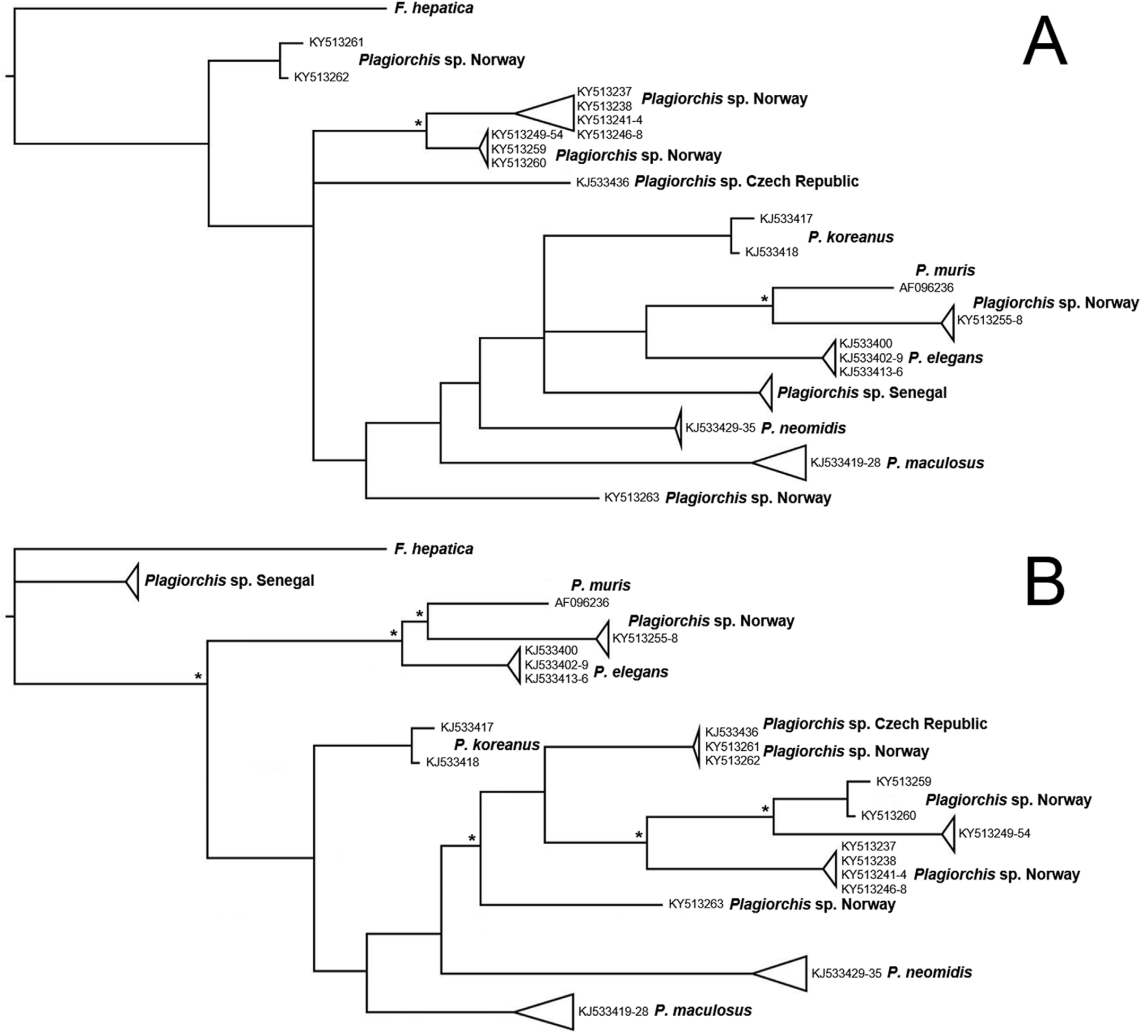
Phylogenetic relationships among *Plagiorchis* spp. inferred by Maximum Likelihood (A) and Bayesian Inference (B) analyses of the cytochrome *c* oxidase subunit 1 gene data. The taxon *Fasciola hepatica* (GenBank™ AP017707) was used as outgroup. Nodal support ≥ 80% from likelihood bootstrap replicates and Bayesian posterior probabilities is indicated with an asterisk.

Sequencing of ITS and *cox1* regions (1159 and 396 base pairs, respectively) identified one distinct *Plagiorchis* species, with no intraspecific variation within the ITS, while pairwise comparison of the *cox1* sequences showed ≥ 99.2% similarity independent of host species and/or locality. Sequencing of individual cercariae showed the presence of the same *Plagiorchis* sp. in one *R. natalensis* collected in Merina Guewel (15°56′38.9″N, 15°58′19.1″W), a sampling site within LG_3. The ITS alignment excluded 314 of 1243 sites based on posterior probability filtering. ML analysis of the resulting ITS dataset (929 characters) yielded a single best-scoring tree strongly supporting monophyly of our West African *Plagiorchis* lineage (including isolates from *M. huberti*, *A. niloticus*, *Crocidura* sp., and *R. natalensis*). ML bootstrap values and BI posterior probabilities yielded a highly resolved topology indicating two clades, one composed by *Plagiorchis* spp. infecting rodents, insectivores, and birds, while the other included parasites of bats (Fig. 2). In contrast, ML and BI analyses of the *cox1* alignment (396 characters, no internal gaps) failed to resolve relationships between our West African lineage and other *Plagiorchis* taxa (Fig. 3).

The binomial GLM showed a significant association (*P* < 0.001) between probability of infection and host species (lower in *A. niloticus*), sampling areas (higher in LG_3), and adult age, whereas host gender did not show a significant effect on *Plagiorchis* sp. prevalence. Both the hepatic system and gastrointestinal tract were examined for a subset of 431 individuals (i.e., 288 *M. huberti*, 129 *A. niloticus*, and 14 *Crocidura* sp.); higher infection prevalence (χ^2^ = 54.5; d.f. = 1; *P* < 0.001) and intensity (*P* < 0.001) were found in the biliary tract (the analysis also yielded *P* < 0.001 when we excluded those hosts for which intensity counts stopped at 61), with only eight *M. huberti* (4.5%) harbouring *Plagiorchis* sp. exclusively in the small intestine (intensity range 1–9, median 2).

Adults of *Plagiorchis* sp. were deposited in the collection of the Natural History Museum (London, UK) under the accession numbers 2018.3.7.39–67. Sequencing data were deposited in the GenBank™ database under the accession numbers MH633855-62 (ITS) and MH673675-82 (*cox1*).

## 4 Discussion

This study provides molecular data to characterise a previously undescribed *Plagiorchis* sp. and resolve its phylogenetic relationships within the genus. Sequencing of the ITS region proved to be useful for characterising *Plagiorchis* spp. (Tkach et al., 2000; Boyce et al., 2014), while the simultaneous examination of rDNA and mtDNA loci is suitable for molecular prospecting and further hypothesis testing on species delimitation (Nadler and Pérez-Ponce de León, 2011). Our findings revealed that *Plagiorchis* isolates from *M. huberti*, *A. niloticus*, and *Crocidura* sp., represented the same evolutionary lineage. Furthermore, we recorded the occurrence of this parasite in one freshwater gastropod *R. natalensis*, which may act as a suitable first intermediate host in the region of Lake Guiers. The phylogenetic analysis indicated that an unidentified *Plagiorchis* sp. found in the caddisfly genus *Lepidostoma* (GenBank™ KX160474) was most closely related to our West African lineage. However, the identity of this parasite specimen is not confirmed and is hypothesized to be *Plagiorchis neomidis*, a species described from the Eurasian water shrew (*Neomys fodiens*) (Brendow, 1970), according to its phylogenetic position in Zikmundová et al. (2014). Within the same clade of our West African *Plagiorchis* sp., the most closely related identified species were *Plagiorchis maculosus*, a parasite of insectivorous passerines and other birds (e.g., Bušta, 1987), and *Plagiorchis elegans*, a common parasite of rodents, various mammalian hosts, and birds of the Northern Hemisphere (e.g., Boyce et al., 2014). Therefore, our results supported two divergent lineages within the *Plagiorchis* genus, one of which appears restricted to chiropterans as definitive hosts. Bats, rodents, and insectivores may have acquired the ancestors of their modern *Plagiorchis* spp. from birds (Tkach et al., 2000), and the phylogenetic structure we observed may be the result of the ancient evolutionary histories and ecological differences between these mammalian orders. Nevertheless, our understanding of the genus *Plagiorchis* is poor and molecular data for further comparisons are lacking; this is evidenced by the scarce phylogenetic resolution for *P. muris*, the only species within the rodent clade for which human infections have been documented (Asada et al., 1962; Hong et al., 1996). However, the trees generated from the *cox1* dataset partially supported isolates of *P. elegans*, and of an unidentified *Plagiorchis* sp. from Norway, as closely related to the potentially zoonotic *P. muris*. Relatedness between *P. elegans* and *P. muris* is also suggested by morphological studies, which documented the high degree of similarity between the two *Plagiorchis* spp. and even hypothesized them as synonyms for the same species (Boyce et al., 2014).

The high prevalence and intensity of *Plagiorchis* sp. in the examined mice and shrews, including its occurrence in Nile grass rats, confirmed the wide host spectrum of this parasite and its ubiquitous presence in the region of Lake Guiers, particularly in the sampling area LG_3. Moreover, we found a significant increase in *Plagiorchis* sp. prevalence with host age. While this might be driven by our sampled population predominantly composed by *M. huberti*, the observed pattern is consistent with a higher probability of exposure to *Plagiorchis* infective stages as host age increases, as has been observed in England for infections by *P. elegans* in the wood mouse (*Apodemus sylvaticus*) (Boyce et al., 2014). In contrast, small mammals from the RT_1 area were not found to be infected, which might be due to different first and/or second intermediate host densities and other ecological processes affecting the life cycle of *Plagiorchis* sp., rather than to the local abundance of definitive hosts. Additional field data are needed to identify the species of freshwater snails and second intermediate hosts that facilitate the transmission of *Plagiorchis* sp., and whether these hosts could be potential determinants of exposure, especially for humans. Nevertheless, our results indicated that *M. huberti* might play a primary role in the epidemiology of the parasite locally. Geographical distribution, transmission patterns, and potential zoonotic risk of *Plagiorchis* sp. in West Africa could be driven by a strong association with humid zones (e.g., riparian habitats and irrigated agricultural settlements), which are the exclusive environment types occupied by *M. huberti* (Mouline et al., 2008; Granjon and Duplantier, 2009). Therefore, we speculate that dipteran larvae, which thrive in freshwater ecosystems, may act as potential hosts for *Plagiorchis* sp. in northern Senegal because insects are prevalent in the diet of *M. huberti* and *Crocidura* spp., whereas the lower infection probability in *A. niloticus* could be reflected by the dietary preferences of this rodent species, which predominantly feeds on seeds and vegetation (Granjon and Duplantier, 2009). A similar hypothesis has been made by Boyce et al. (2014), with apparent positive association between the presence of insect remains in the stomach contents of wood mice harbouring *P. elegans*. Insects play a significant role as a second intermediate host for the zoonotic *P. vespertilionis* and *P. muris* too; however, the consumption of raw freshwater fish was regarded as a plausible source of infection in human cases (Hong et al., 1996; Guk et al., 2007). Whether freshwater fish also act as second intermediate hosts for *Plagiorchis* sp. in the region of Lake Guiers is unknown, but the central role of fish in the diet of Senegalese communities poses a potential risk of zoonotic food-borne trematodiases. Moreover, our findings strongly indicate the biliary tract as the final destination of *Plagiorchis* sp., as opposed to the small intestine which is described to be the classical site of infection within the definitive host (e.g., Guk et al., 2007; Boyce et al., 2014). The histopathological changes of the biliary tract observed during our study closely resembled the chronic nature and lesions caused by the trematodes *Clonorchis sinensis* and *Opistorchis* spp. in humans and other mammalian hosts (Sripa et al., 2010). If zoonotic cases were demonstrated, *Plagiorchis* sp. may be an alternative aetiological agent of the extensive pathological changes we observed in the hepatic system of human patients, sometimes independent of their infection status with schistosomiasis disease, during ultrasonography in the same localities (our unpublished data). Thus, the potential role of *Plagiorchis* sp. as an emerging food-borne trematodiasis in the region of Lake Guiers warrants further investigations. Anthropogenic alterations of the Senegal River Basin after the construction of the Diama Dam include the plan to restore populations of the river prawn *Macrobrachium vollenhoveni* for the biological control of freshwater snails transmitting schistosomiasis and other trematodiases (Sokolow et al., 2015; Jones et al., 2018). However, this approach may inadvertently favour the establishment of *Plagiorchis* parasites since malacostracan crustaceans could potentially act as second intermediate hosts (Soldánová et al., 2017).

## 5 Conclusions

In conclusion, verification of the zoonotic potential of this newly discovered, multi-host *Plagiorchis* sp. will require further diagnostic and epidemiological studies. Our data suggest that major drivers influencing the success of host colonization (i.e., opportunity and compatibility to initiate exploitation of new hosts; see Araujo et al., 2015) have the potential to be met. With the exception of *Schistosoma* spp., which infect humans percutaneously, most medically important trematodes are food-borne parasites acquired through the consumption of fish, crustaceans, and aquatic plants harbouring infective parasitic larvae (Keiser and Utzinger, 2005; Pérez-Ponce de León and Nadler, 2016). Infection risk estimates for communities residing near freshwater bodies, in addition to the recognition of the public health significance of zoonotic trematodiases and their link to poverty and land-use change, are increasing, with millions of people globally suffering one or more food-borne trematodiases (Keiser and Utzinger, 2005, 2009). The detection and characterisation of parasite species in a proactive manner, by delimiting geographical areas, identifying animal reservoirs, and evaluating zoonotic potential are key components to integrated control strategies (Pérez-Ponce de León and Nadler, 2016). The ubiquity of *Plagiorchis* sp. at a local level is clear; therefore, we need to understand the host community in which this parasite is embedded in order to facilitate interventions and more effective management geared to enabling people, domestic animals, and wildlife to coexist with a reduced disease transmission risk in the Senegal River Basin and West Africa as a whole.

## Declarations of interest

None.

## Note

Supplementary data associated with this article.

## Appendix A Supplementary data

PLAGIOijppaw_supplmat_FINAL.docx PLAGIOijppaw_supplmat_FINAL.docx
