# Supplementary material for: Plagiorchis sp. in small mammals of Senegal and the potential emergence of a zoonotic trematodiasis
Source: Int J Parasitol Parasites Wildl. 2019 Feb 14;8:164–70. doi: 10.1016/j.ijppaw.2019.02.003 (PMC6384303; doi:10.1016/j.ijppaw.2019.02.003)
Supplement: PLAGIOijppaw_supplmat_FINAL.docx [file mmc1.docx]

**SUPPLEMENTARY DATA**

***Plagiorchis* sp. in small mammals of Senegal and the potential emergence of a zoonotic trematodiasis**

S. Catalano, S.A. Nadler, C.B. Fall, K.J. Marsh, E. Léger, M. Sène, S.L. Priestnall, C.L. Wood, N.D. Diouf, K. Bâ, and J.P. Webster

Correspondence: S. Catalano, Centre for Emerging, Endemic and Exotic Diseases, Department of Pathobiology and Population Sciences, The Royal Veterinary College, University of London, Hatfield, AL9 7TA, United Kingdom (scatalano@rvc.ac.uk).

**Supplementary Fig. S1.** Map of the sampling sites in northern Senegal. Triangles indicate the localities surveyed in and around the town of Richard Toll (RT_1), while the sampling sites on the shores of Lake Guiers are represented by stars (LG_1), circles (LG_2), and squares (LG_3). Base layer is Google Satellite (2019).


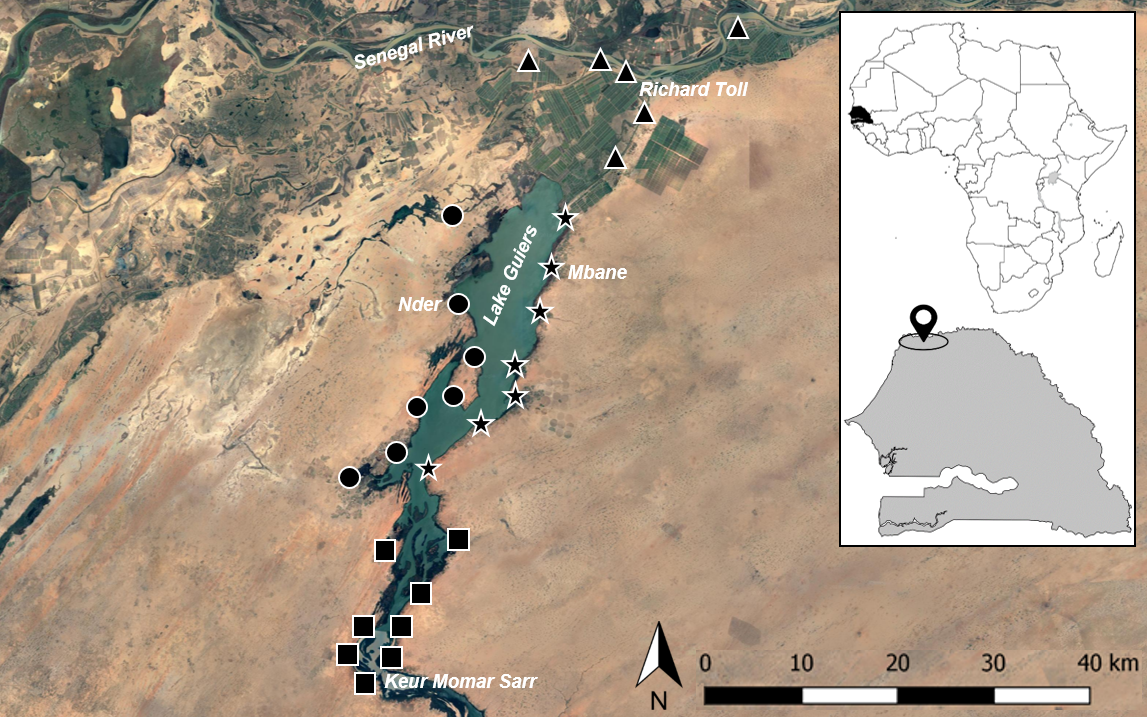


**Supplementary Table S1.** Universal Transverse Mercator (UTM) coordinates and habitat types at each sampling site within four areas in and around the town of Richard Toll (RT_1), and on the shores of Lake Guiers (LG_1, LG_2, and LG_3), Senegal.

| **Sampling site** | **UTM coordinates** | | **Habitat type** | |
| --- | --- | --- | --- | --- |
| **RT_1 sites** | | | |  |
| **Alarba** | 28Q 424944 1817675 | | Agricultural land | |
| **Djidiery** | 28Q 434537 1822242 | | Agricultural land and riparian habitat | |
| **Rosso** | 28Q 414018 1820608 | | Riparian habitat | |
| **RT canal** | 28Q 423711 1821079 | | Agricultural land | |
| **RT river** | 28Q 423611 1821614 | | Riparian habitat | |
| **Thiago** | 28Q 422887 1813134 | | Agricultural land | |
| **LG_1 sites** | |  | |  |
| **Foss** | 28Q 409514 1786812 | | Riparian habitat | |
| **Guidick** | 28Q 403218 1781600 | | Agricultural land and shrubs | |
| **Mbane** | 28Q 414315 1799080 | | Agricultural land and riparian habitat | |
| **Ndiakhaye** | 28Q 411879 1790310 | | Riparian habitat | |
| **Pomo** | 28Q 413482 1793292 | | Riparian habitat | |
| **Saneinte** | 28Q 414316 1795777 | | Shrubs and riparian habitat | |
| **Temey** | 28Q 417538 1806038 | | Riparian habitat | |
| **LG_2 sites** | |  | |  |
| **Diokhor** | 28Q 405650 1790070 | | Riparian habitat | |
| **Malla** | 28Q 401755 1785376 | | Riparian habitat | |
| **Nder** | 28Q 406603 1798615 | | Riparian habitat | |
| **Ndieumeul** | 28Q 408200 1793208 | | Riparian habitat | |
| **Ngnith** | 28Q 402619 1788643 | | Agricultural land and riparian habitat | |
| **Yamane** | 28Q 394854 1780516 | | Shrubs and riparian habitat | |
| **Yetti-Yone** | 28Q 405721 1807693 | | Riparian habitat | |
| **LG_3 sites** | |  | |  |
| **Diaminar** | 28Q 403440 1770923 | | Riparian habitat | |
| **Feto** | 28P 396044 1762392 | | Riparian habitat | |
| **Ganket** | 28P 400370 1767221 | | Shrubs and riparian habitat | |
| **Gueo** | 28P 398984 1762405 | | Shrubs and riparian habitat | |
| **Keur Momar Sarr** | 28P 396747 1761044 | | Riparian habitat | |
| **Mbrar** | 28Q 400616 1773916 | | Riparian habitat | |
| **Merina Guewel** | 28P 395943 1762998 | | Riparian habitat | |
| **Syere** | 28Q 404176 1777690 | | Riparian habitat | |

**Supplementary Table S2.** Ratio between infected and negative hosts with *Plagiorchis* sp. (intensity median and range in parentheses) captured at each sampling site within four areas in and around the town of Richard Toll (RT_1), and on the shores of Lake Guiers (LG_1, LG_2, and LG_3), Senegal. The capture rate represents the proportion between captures and number of active traps set overnight.

| **Sampling site** | | **Capture rate** | **Organ** | ***Arvicanthis niloticus*** | | ***Mastomys huberti*** | | ***Crocidura* sp.** | ***Taterillus* sp.** |
| --- | --- | --- | --- | --- | --- | --- | --- | --- | --- |
|  |  |  |  | **Juveniles** | **Adults** | **Juveniles** | **Adults** |  |  |
| **RT_1 sites** | | | | | | | | | |
| **Alarba** |  | 17.1% | Liver | 0/7 | 0/21 | 0/1 | 0/6 | 0/5 | 0/3 |
|  |  |  | Intestine | 0/2 | 0/14 | - | 0/6 | - | - |
| **Djidiery** |  | 21.0% | Liver | 0/26 | 0/43 | 0/5 | 0/7 | 0/4 | 0/3 |
|  |  |  | Intestine | 0/3 | 0/16 | 0/2 | 0/5 | - | - |
| **Rosso** |  | 40.0% | Liver | 0/4 | 0/2 | 0/6 | 0/4 | - | - |
|  |  |  | Intestine | - | - | 0/1 | - | - | - |
| **RT canal** |  | 14.5% | Liver | 0/25 | 0/48 | - | 0/2 | 0/5 | - |
|  |  |  | Intestine | 0/9 | 0/27 | - | 0/2 | - | - |
| **RT river** |  | 4.0% | Liver | - | - | 0/4 | 0/4 | 0/2 | - |
|  |  |  | Intestine | - | - | 0/3 | 0/4 | - | - |
| **Thiago** |  | 8.7% | Liver | - | - | 0/3 | 0/1 | 0/3 | - |
|  |  |  | Intestine | - | - | 0/2 | 0/1 | - | - |
| **LG_1 sites** | | | | | | | | | |
| **Foss** |  | 26.7% | Liver | - | 0/5 | - | 4/7 (11.5, 3-49) | 1/4 (>61) | - |
|  |  |  | Intestine | - | 0/5 | - | 2/7 (2-6) | 0/4 | - |
| **Guidick** |  | 0% | Liver | - | - | - | - | - | - |
|  |  |  | Intestine | - | - | - | - | - | - |
| **Mbane** |  | 26.5% | Liver | 0/7 | 0/27 | 1/6 (3) | 28/54 (16, 1->61) | 4/9 (46, 5->61) | - |
|  |  |  | Intestine | 0/1 | 0/10 | 0/4 | 4/31 (4, 1-17) | 0/1 | - |
| **Ndiakhaye** |  | 11.7% | Liver | - | 0/1 | - | 3/5 (1, 1-18) | 0/1 | - |
|  |  |  | Intestine | - | 0/1 | - | 0/5 | 0/1 | - |
| **Pomo** |  | 10.0% | Liver | - | - | 0/1 | 3/4 (2, 2->61) | 1/3 (57) | - |
|  |  |  | Intestine | - | - | 0/1 | 1/4 (>61) | 0/3 | - |
| **Saneinte** |  | 7.5% | Liver | - | 0/2 | - | 1/1 (2) | - | - |
|  |  |  | Intestine | - | 0/2 | - | 0/1 | - | - |
| **Temey** |  | 24.1% | Liver | - | 0/4 | 3/12 (18, 5-26) | 22/31 (21.5, 1->61) | 1/5 (>61) | - |
|  |  |  | Intestine | - | 0/4 | 1/8 (19) | 8/28 (4.5, 1-21) | 0/5 | - |
| **LG_2 sites** | | | | | | | | | |
| **Diokhor** |  | 13.8% | Liver | - | 0/2 | 0/3 | 6/13 (6.5, 1->61) | - | - |
|  |  |  | Intestine | - | 0/2 | 0/3 | 3/13 (4, 3-5) | - | - |
| **Malla** |  | 7.5% | Liver | - | 0/2 | - | 3/4 (>61, 2->61) | - | - |
|  |  |  | Intestine | - | 0/2 | - | 0/4 | - | - |
| **Nder** |  | 18.9% | Liver | 0/1 | 2/10 (1-37) | 13/24 (30, 3->61) | 18/36 (22.5, 3->61) | - | - |
|  |  |  | Intestine | 0/1 | 0/10 | 0/9 | 2/23 (2-9) | - | - |
| **Ndieumeul** |  | 17.7% | Liver | - | 2/3 (1-2) | 1/4 (5) | 7/16 (5, 1->61) | - | - |
|  |  |  | Intestine | - | 0/3 | 0/4 | 1/16 (26) | - | - |
| **Ngnith** |  | 20.8% | Liver | - | 0/10 | 0/2 | 4/13 (6.5, 2-21) | - | - |
|  |  |  | Intestine | - | 0/10 | 0/2 | 3/13 (1, 1-9) | - | - |
| **Yamane** |  | 0% | Liver | - | - | - | - | - | - |
|  |  |  | Intestine | - | - | - | - | - | - |
| **Yetti-Yone** |  | 8.9% | Liver | - | - | 0/3 | 1/5 (29) | - | - |
|  |  |  | Intestine | - | - | 0/3 | 1/3 (1) | - | - |
| **LG_3 sites** | | | | | | | | | |
| **Diaminar** |  | 8.9% | Liver | - | - | 1/1 (>61) | 3/4 (>61, >61->61) | - | - |
|  |  |  | Intestine | - | - | 1/1 (8) | 2/4 (5-12) | - | - |
| **Feto** |  | 18.3% | Liver | - | 0/1 | 4/5 (>61, 21->61) | 5/5 (>61, 43->61) | - | - |
|  |  |  | Intestine | - | 0/1 | 1/5 (2) | 1/5 (3) | - | - |
| **Ganket** |  | 10.8% | Liver | - | 1/4 (>61) | - | 3/4 (24, 17-26) | - | - |
|  |  |  | Intestine | - | 0/4 | - | 3/4 (6, 4-9) | - | - |
| **Gueo** |  | 14.8% | Liver | - | - | 2/4 (19-46) | 14-15 (>61, 1->61) | - | - |
|  |  |  | Intestine | - | - | 1/4 (5) | 13/15 (9, 1-29) | - | - |
| **Keur Momar Sarr** |  | 27.1% | Liver | - | - | 6/7 (27.5, 13->61) | 12/12 (>61, 12->61) | - | - |
|  |  |  | Intestine | - | - | 2/7 (2-8) | 11/12 (18, 2->61) | - | - |
| **Mbrar** |  | 20.0% | Liver | - | 0/2 | 0/5 | 5/5 (19, 8->61) | - | - |
|  |  |  | Intestine | - | 0/2 | 1/5 (1) | 3/5 (11, 1-17) | - | - |
| **Merina Guewel** |  | 17.6% | Liver | - | - | 1/3 (7) | 6/9 (>61, 8->61) | - | - |
|  |  |  | Intestine | - | - | 0/3 | 4/9 (6.5, 2-34) | - | - |
| **Syere** |  | 2.5% | Liver | - | - | - | 1/1 (>61) | - | - |
|  |  |  | Intestine | - | - | - | 1/1 (2) | - | - |
